# Supplementary material for: The genome sequence of the Common Goldeneye, Bucephala clangula (Linnaeus, 1758)
Source: Wellcome Open Res. 2025 May 20;10:258. [Version 1] doi: 10.12688/wellcomeopenres.24275.1 (PMC12209769; doi:10.12688/wellcomeopenres.24275.1)
Supplement: Peer review history

# Reviewer report: Reviewer response for version 1

**DOI:** 10.21956/wellcomeopenres.26773.r125092

**Published:** 2025-06-30

**Authors:** Martin Pippel

**Affiliations:** 1 Department of Cell and Molecular Biology, Uppsala University, Husargatan 3, Uppsala, Uppsala, Sweden

“The genome sequence of the Common Goldeneye, *Bucephala clangula *(Linnaeus, 1758)” from Colom *et al. *describes a high-quality genome assembly based on PacBio HiFi reads and Hi-C data. 

 The data note is well structured and the writing is clear and concise. The background information provides a brief insight about the Common Goldeneye. With the described methods and the publicly available sequencing data the assembly should be reproducible by the scientific community.

 Methods:

 Sample and library preparation are described in great detail, whereas the assembly part falls a bit short. For a genome that possesses microchromosomes it would be nice to make use of the tool MicroFinder ( https://github.com/sanger-tol/MicroFinder) in order to identify and assign smaller contigs with mito genes to putative microchromosomes. Furthermore, program arguments of the used tools are not described. Even if all tools were run in default mode, it would be worth mentioning.

 I do only have some minor comments and suggestions:

  

- Table 1: accession ERR12257416 has a read and a base count of 0.  ENA only provides a 18Gb bam file for this accession. But, does this data set even belong to the Common Goldeneye and was it used in the assembly?
- Table 2 and text: The final primary assembly consists of 537 scaffolds. But the accession GCA_964059595.1 lists 538 scaffolds. I assume this is due to adding the mitochondrial genome afterwards.
- Figure 3: the interactive version is not available, the phylum is not plotted in the figure – it is just labelled as blank
- Figure 4: the interactive cumulative sequence plot links to the snail plot (Figure 2), the phylum is not plotted in the figure – it is just labelled as blank
- Assembly:
  - A k-mer database was generated from filtered reads using FastK. How and why were the reads filtered?
  - Yahs was used with the –break option. To my knowledge there is no break option in yahs. By default yahs is doing a contig and a scaffold error correction. The contigs were indeed split into smaller pieces which helps to correct for errors in the contigging. On the other hand yahs can also oversplit contigs. In this Common Goldeneye assembly 25 contigs are smaller or equal to 5 Kb which were trimmed off by yahs from larger contigs. Usually the Hi-C sequencing coverage (i.e. the resolution) is not high enough to support cuts of these short lengths. In some cases contig ends that show telomere motifs are trimmed off – this probably happened for CAXKXP010000212.1 and CAXKXP010000339.1
- Two different spellings for: MERQUERY.FK and Merquery.FK
- Table 4:
  - PretextSnapShot: missing version
  - FastK: I could not find the provided version
  - HiGlass: I could not find the provided versions

Are sufficient details of methods and materials provided to allow replication by others?

Yes

Is the rationale for creating the dataset(s) clearly described?

Yes

Are the datasets clearly presented in a useable and accessible format?

Yes

Are the protocols appropriate and is the work technically sound?

Yes

Reviewer Expertise:

Genome assembly and annotation, pipeline development

I confirm that I have read this submission and believe that I have an appropriate level of expertise to confirm that it is of an acceptable scientific standard.

# Reviewer report: Reviewer response for version 1

**DOI:** 10.21956/wellcomeopenres.26773.r124392

**Published:** 2025-06-19

**Authors:** Rauri C.K. Bowie

**Affiliations:** 1 University of California Berkeley, Berkeley, California, USA

This paper utilizes PacBio and Hi-C sequencing to generate a highly contiguous genome assembly for the Common Goldeneye. The study is well designed and will serve as an excellent genomic resource for the research community.

**Background**

 When introducing the species it would be helpful to add a map depicting the distribution range of the species.

 “ *The male’s eyes stand out due to its dark green (almost black) head, white cheeks, and black-and-white striped wings.” *Point out the white cheek is a circle which distinguishes this species from its close relative Barrow’s Goldeneye ( *B.  islandica*) which has a sickle shaped white patch on the cheek.

 4 ^th^ paragraph under Background: Anatidae comprises both diving and dabbling ducks so it is more accurate to state that they can forage to depths of 7 m.  The word “to” is missing.

 I understand there may be a reason for the chosen format, but it would have been easier to follow if the Methods section had preceded the Genome Sequence Report. While reviewing, I repeatedly noted the need for additional information, which I later found in the

**Methods**

 Under 'Sequencing Data,' it may be clearer to state '45X coverage' rather than just '45 coverage.' It would also be helpful to specify which tissues were used for RNA-seq data collection. In the Methods section, I only noted muscle—was this the only tissue sequenced?

 Under 'Assembly Quality Metrics' – What constitutes a good QV (overall quality) value? The general reader would benefit from guidance on how to interpret this metric, or alternatively, you could provide the typical range of values it can span.

 Sample acquisition – Please check that the voucher specimen number *NHMUK014561651* is correct as it does to seem typical of the NHM catalogue numbers.

 Hi-C Sample preparation and crosslinking - “restriction enzyme master mix” – specify the restriction enzymes used.

 Mitochondrial genome assembly:  for MitoHiFi was a guide mtDNA genome used?  If so, please detail this and provide the NCBI accession number.

 Figures 3 and 4 do not add much and can be omitted from the main text and moved to the sup docs.

**Data availability**

*Bucephala clangula* should be in italics.

Are sufficient details of methods and materials provided to allow replication by others?

Yes

Is the rationale for creating the dataset(s) clearly described?

Yes

Are the datasets clearly presented in a useable and accessible format?

Yes

Are the protocols appropriate and is the work technically sound?

Yes

Reviewer Expertise:

Evolutionary Biology, Ornithology

I confirm that I have read this submission and believe that I have an appropriate level of expertise to confirm that it is of an acceptable scientific standard.
